# Retraction: Application of magnetite nano-hybrid epoxy as protective marine coatings for steel

**DOI:** 10.1039/d5ra90073e

**Published:** 2025-06-09

**Authors:** Ayman M. Atta, Ashraf M. El-Saeed, Gamal M. El-Mahdy, Hamad A. Al-Lohedan

**Affiliations:** a Surfactants Research Chair, Chemistry Department, College of Science, King Saud University Riyadh 11451 Saudi Arabia aatta@ksu.edu.sa hlohedan@ksu.edu.sa +966 114675998 +966 561557975; b Petroleum Application Department, Egyptian Petroleum Research Institute Nasr City 11727 Cairo Egypt ashrfelsaied@yahoo.com +20 222747433 +20 222747917; c Chemistry Department, Faculty of Science, Helwan University Helwan Egypt

## Abstract

Retraction of ‘Application of magnetite nano-hybrid epoxy as protective marine coatings for steel’ by Ayman M. Atta *et al.*, *RSC Adv.*, 2015, **5**, 101923–101931, https://doi.org/10.1039/C5RA20730D.

The Royal Society of Chemistry wholly retracts this *RSC Advances* article due to concerns with the reliability of the data.

The XRD pattern in Fig. 2, the HR-TEM data in Fig. 3 and the SEM data in Fig. 4a and b all contain repeating sections.

The authors were not able to provide the original raw data nor satisfactorily explain the concerns.

Given the significance of these concerns, the findings presented in this paper are no longer reliable.

The authors were informed of the decision to retract. Ayman M. Atta has not agreed with the decision, the other authors have not responded.

Signed: Laura Fisher, Executive Editor, *RSC Advances*

Date: 28th May 2025

